# Comparative analysis of NOAA REFM and SNB^3^GEO tools for the forecast of the fluxes of high‐energy electrons at GEO

**DOI:** 10.1002/2015SW001303

**Published:** 2016-01-28

**Authors:** M. A. Balikhin, J. V. Rodriguez, R. J. Boynton, S. N. Walker, H. Aryan, D. G. Sibeck, S. A. Billings

**Affiliations:** ^1^ ACSEUniversity of SheffieldSheffieldUK; ^2^ Cooperative Institute for Research in Environmental SciencesUniversity of Colorado BoulderBoulderColoradoUSA; ^3^ National Centers for Environmental InformationNational Oceanic and Atmospheric AdministrationBoulderColoradoUSA; ^4^ GSFCNASAGreenbeltMarylandUSA

**Keywords:** radiation belt forecast

## Abstract

Reliable forecasts of relativistic electrons at geostationary orbit (GEO) are important for the mitigation of their hazardous effects on spacecraft at GEO. For a number of years the Space Weather Prediction Center at NOAA has provided advanced online forecasts of the fluence of electrons with energy >2 MeV at GEO using the Relativistic Electron Forecast Model (REFM). The REFM forecasts are based on real‐time solar wind speed observations at L1. The high reliability of this forecasting tool serves as a benchmark for the assessment of other forecasting tools. Since 2012 the Sheffield SNB^3^GEO model has been operating online, providing a 24 h ahead forecast of the same fluxes. In addition to solar wind speed, the SNB^3^GEO forecasts use solar wind density and interplanetary magnetic field *B*_*z*_ observations at L1.The period of joint operation of both of these forecasts has been used to compare their accuracy. Daily averaged measurements of electron fluxes by GOES 13 have been used to estimate the prediction efficiency of both forecasting tools. To assess the reliability of both models to forecast infrequent events of very high fluxes, the Heidke skill score was employed. The results obtained indicate that SNB^3^GEO provides a more accurate 1 day ahead forecast when compared to REFM. It is shown that the correction methodology utilized by REFM potentially can improve the SNB^3^GEO forecast.

## Key Points

- Accuracy of the electron fluence forecast by REFM and SNB3GEO is assessed using GOES 13 data
- Prediction efficiency, correlation, and Heidke skill scores indicate higher accuracy of SNB^3^GEO
- The data correction methodology used by REFM may improve SNB^3^GEO accuracy even further

## 1 Introduction

The smooth operation of modern society depends upon the ability for the fast exchange of information between various organizations and individuals. Spacecraft operating at geostationary orbit (GEO) form an essential part of our modern technological infrastructure that enables this information exchange. Adverse space weather conditions may result in the interruption of services provided by spacecraft at GEO, thereby leading to a reduction, temporary cessation, or possibly termination of the communication/navigational services used in many aspects of modern society. In spite of the provision of some shielding on board the spacecraft, energetic electrons are still able to penetrate deep inside the spacecraft subsystems and components, potentially resulting in internally induced electrostatic discharges. Internally induced electrostatic discharges can cause anomalies in spacecraft operation and even the failure of vulnerable subsystems [Vampola, 1987; *Baker et al.*, 1987; Wrenn, 1995; *Fennell et al.*, 2000; Bodeau, 2010]. Thus, the reliable forecast of high‐energy electron fluxes at GEO can assist in the mitigation of hazardous space weather effects on spacecraft operations there.

In the current paper, the quality of the forecasts of daily averaged fluxes of energetic electrons (>2 MeV) at GEO by the Relativistic Electron Forecast Model (REFM) and SNB^3^GEO tools is assessed. The forecast of >2 MeV electron fluxes at GEO from three models [REFM *Baker et al.*, 1990, Li, 2004 and FluxPred Ling, 2000; *Ling et al.*, 2010] have been compared by *Perry et al.* [2010] together with persistence and reoccurrence models. [*Perry et al.*, 2010] demonstrated that all of the models had clear solar cycle dependencies and concluded that on average the models perform very similarly. As it was stated in *Perry et al.*[2010] “After evaluating all the models, there was no clear winner; each model did well at different phases of the solar cycle.” The present work compares the benchmark REFM with SNB^3^GEO, which did not operate when *Perry et al.* [2010] was published. The paper is organized as follows. Section 2 reviews the REFM and SNB^3^GEO tools as well as the approaches used in their development. Section 3 describes the GOES 13 data and methodology employed in the current paper to compare the quality of the forecasts. Section 4 presents the results of this comparison. Section 5 describes that the correction methodology utilized by REFM has a potential to improve the SNB^3^GEO forecast.

## 2 The SNB^3^GEO and REFM Forecast Tools

The Sheffield SNB^3^GEO online forecast tool has been operating continually since 2 March 2012. Its results are available at http://ssg.group.shef.ac.uk/ssg2013/UOSSW/2MeV_EF.html. The Sheffield SNB^3^GEO model was derived using the NARMAX (Nonlinear AutoRegressive Moving Average models with eXogenous input) system identification algorithm [Leontaritis and Billings, 1985a, 1985b].

System identification techniques can automatically derive a model based on data sets that represent the input and output of a system. These are often known as black box methodologies since any knowledge of the processes within the system is not required. It is only necessary to provide data for the external influences on the system (ACE solar wind measurements) and measurements of the output data (GOES 12 electron flux measurements). NARMAX is only one of a number of methodologies that have been developed in systems science to identify unknown dynamical systems. In contrast to some other methodologies such as Neural Networks, NARMAX provides physically interpretable models that can assist in the understanding of the physical processes that underpin the evolution of complex dynamical systems. The NARMAX system identification methodology has been applied to a wide variety of complex systems in many different scientific fields. For example, in biology, *Friedrich et al.* [2009] used the technique to analyze the adaptive changes in the photoreceptors of Drosophila. The identification of the Belousov‐Zhabotinsky chemical reaction was found from cellular automata NARMAX models [*Zhao et al.*, 2007]. In the field of space physics, NARMAX has been used to model the radiation belts [*Balikhin et al.*, 2011; *Boynton et al.*, 2013, 2015] and geomagnetic storms [*Boaghe et al.*, 2001; *Wei et al.*, 2004; *Boynton et al.*, 2011a]. The methodology has also been used to analyze the magnetospheric system. *Boynton et al.* [2011b] used the Error Reduction Ratio (ERR) methodology to determine a coupling function for the Dst index, which was validated by *Balikhin et al.*[2010]. *Boynton et al.* [2013] investigated the solar wind control of the electron fluxes at GEO and determined a relationship between the solar wind parameters and the evolution of the electron flux in various energy ranges. *Balikhin et al.* [2012] compared these results to the energy diffusion equation and determined that local acceleration of electrons was not dominant at GEO.

The NARMAX methodology was first developed by Leontaritis and Billings [1985a, 1985b] and a Forward Regression Orthogonal Least Squares (FROLS) algorithm, which can deduce the model structure and estimate the coefficients, was proposed by *Billings et al.* [1988, 1989]. A comprehensive review of all aspects of NARMAX methodology may be found in Billings [2013]. SNB^3^GEO uses a Multi‐Input Single‐Output (MISO) NARMAX model to represent the dynamics of the electron fluxes at GEO. The forecast at time *t*, ŷ(t), resulting from the general MISO NARMAX model can be represented by equation (1).

(1) $$ŷ(t)=F[y(t-1),\ldots ,y(t-n_{y}),u_{1}(t-1),\ldots ,u_{1}(t-n_{u_{1}}),\ldots ,u_{m}(t-1),\ldots ,u_{m}(t-n_{u_{m}}),\ldots ,e(t-1),\ldots ,e(t-n_{e})]+e(t)$$

where *y*, *u*, and *e* represent the output, input, and error terms, respectively; *m* is the number of system inputs; and *n*_*y*_, $n_{u_{1}},\ldots ,n_{u_{m}}$, *n*_*e*_ are the maximum time lags for the output, each of the *m* inputs, and the error, respectively. In the case of the SNB^3^GEO model the nonlinear function *F*[·] is represented in terms of a second‐degree polynomial. The error term at time *t* − 1 is calculated as the residual of the model, e.g., e(t−1)=y(t−1)−ŷ(t−1). The incorporation of error terms *e* into the NARMAX model intends to account for unobserved inputs and measurement errors.

The NARMAX FROLS algorithm [*Billings et al.*, 1989] is one of the most advanced system identification techniques. The first stage of the algorithm is structure detection. The expansion of equation (1) as a polynomial results in many possible monomials, made up from combinations of the cross‐coupled past inputs and past outputs. Most of these monomials will have virtually no effect on the system. Therefore, the monomials with greatest influence on the system need to be extracted. The NARMAX FROLS algorithm detects these monomials by the use of the ERR. This method is able to find and rank the influence of the monomials that make up the model structure. Once the model structure is detected, the next stage is the coefficient estimation. In this stage, the coefficients of each monomial in the model structure are calculated. The final stage is the validation of the model using nonlinear correlation tests [Billings and Voon, 1986; Billings and Zhu, 1995].

The SNB^3^GEO model was deduced by the FROLS algorithm on data between 11 July 2004 and 11 October 2005 [*Boynton et al.*, 2015]. The input parameters for the model are daily averages of solar wind velocity *v*, density *n*, and the percentage of time that the interplanetary magnetic field (IMF) remains southward within each day $\tau_{B_{s}}$, based on real‐time measurements from the Advanced Composition Explorer (ACE) spacecraft at L1 [*Zwickl et al.*, 1998]. The output data set were daily averaged >2 MeV electron fluxes from GOES 12. The maximum lags employed for the FROLS algorithm were 3 days (includes lags of 1, 2, and 3 days), and the nonlinear function, *F*, was set to be a quadratic polynomial. The resulting NARMAX model was then implemented online and in real time to produce a forecast of >2 MeV electron fluxes. Since the minimum lag for the inputs in the model is 1 day, it is possible to forecast 1 day into the future. For example, at midnight between days *X* and *X* + 1, the averaged solar wind parameters for Day *X* and Day *X* − 1 is calculated using the real‐time ACE data that is downloaded from the Space Weather Prediction Center. Therefore, the model is able to predict the daily averaged flux for the day *X* + 1. This prediction is fixed and transferred to the data archive. It is these predictions that we are use to compare SNB^3^GEO with REFM. At 1 A.M. of the day *X* + 1 the data for solar wind parameters for the time period 00:00 to 01:00 are downloaded. Every hour the model automatically downloads these data (1 min resolution for ACE solar wind and IMF). The mean of the solar wind parameters for the hour 00:00–01:00 h are treated as the mean for the whole day *X* + 1 and are used as model inputs resulting in a preliminary forecast of the daily averaged flux for the day *X* + 2, but this forecast is not fixed. At 02:00, 2 h period of solar wind parameters for Day *X* + 1 is known. It is again treated as the average for the whole day *X* + 1 and an updated preliminary forecast for the day *X* + 2 appear on the model website. This procedure repeats every hour, until midnight from *X* + 1 to *X* + 2. At this moment it is possible to calculate the real value of the averaged solar wind parameters for the day *X* + 1 and forecast for the day *X* + 2 becomes fixed and transferred to the forecast archive. Only the daily averaged fixed forecasts, downloaded from the SNB^3^GEO archive, are used in this study. The model structure and coefficients are beyond the scope of this paper, but this will be addressed by the following publication.

The REFM, running at the Space Weather Predicton Center (SWPC) since 1996, was developed by C. Smithtro based on the linear prediction filter (LPF) method of *Baker et al.* [1990], which predicts radiation belt electron flux in some energy band based on the solar wind speed at some time in the past. In this model, the relationship between some input quantity and the output electron flux is modeled as a convolution of the input quantity with a prediction filter function that is derived from historical comparisons of the input and output data. For the LPF prediction of radiation belt electron fluxes, input quantities such as geomagnetic indices (*K* *p* and *A* *E*) or solar wind velocity have commonly been used [Nagai, 1988; *Baker et al.*, 1990].

REFM calculates 1, 2, and 3 day forecasts of the daily >2 MeV electron fluence at GEO from observations of solar wind velocity at L1, currently from ACE. The prediction filter is derived from 30 days of solar wind velocity. Although the correlation of radiation belt fluxes with solar wind velocity is well established [Paulikas and Blake, 1979], it is unable to account for all observed variability [*Blake et al.*, 1997; *Reeves et al.*, 2011; *Li et al.*, 2011], including short‐term (within 1 day) orders‐of‐magnitude decreases or increases in the flux. In order to improve the short‐term predictions, REFM calculates a flux offset based on recent comparisons between forecasts and daily observations. The 1 day ACE forecast uses the most recent forecast and observation while the 2 and 3 day forecasts use comparisons based on the previous 20 days. For more details on REFM, please see http://www.swpc.noaa.gov/products/relativistic-electron-forecast-model.

## 3 Data and Forecast Assessment Methodology

As mentioned above, SNB^3^GEO has been operating since 2 March 2012 and has generated a continuous set of forecasts from this date. An archive of forecasts is available at http://www.ssg.group.shef.ac.uk/ USSW/Archive_EF/All/All_EF.html from which both graphical and tabular formats of the results are available. REFM has been operating since 1996. The most recent REFM 1, 2, and 3 day ahead forecasts are available in text form from http://services.swpc.noaa.gov/text/relativistic-electron-fluence-tabular.txt.

For the purposes of the comparison, forecasts from the two models were compared for the period from 2 March 2012 until 1 January 2014. During this period the SNB^3^GEO model had a continuous record of forecasts. However, the REFM model output file provided by SWPC for this study contains 10 days for which no forecast is available. Thus, the comparison is based on 661 forecasts during this period when there were predictions from both models.

The measurements with which the forecasts are compared come from the >2 MeV channel of the westward looking Energetic Proton Electron and Alpha Detector (EPEAD) instrument [Hanser, 2011] on the GOES 13 satellite. There are two EPEADs on each GOES, one looking westward and one looking eastward. Since the SWPC forecast office uses the observations from the westward looking EPEAD, we compare the model results with the westward observations. The data for this can be accessed as sets of Daily Particle Data (DPD) files from ftp://ftp.swpc.noaa.gov/pub/indices/old_indices/.

The correlation function and prediction efficiency have been used to assess the accuracy of the 1 day ahead predictions provided by the REFM and SNB^3^GEO tools. The concept of prediction efficiency is based on the model efficiency [e.g., Nash and Sutcliffe, 1970; *Moriasi et al.*, 2007; *Clauer et al.*, 1981; Nagai, 1988]. The prediction efficiency, which estimates the difference between the forecast (*Y*) and measured (*X*) data sets, is defined as

$$\text{PE}=1-\frac{1}{N}\sum \frac{{\left(X_{i}-Y_{i}\right)}^{2}}{\mathrm{Var}(X)}$$

where *N* is the number of points in each data set, $\mathrm{Var}(X)=\frac{1}{N}\sum {\left(X_{i}-〈X_{i}〉\right)}^{2}$, and 〈…〉 is the ensemble average of the parameters. It should be noted that as the discrepancy between forecast and measured values increases the value of PE decreases and may be less than zero. In the ideal case of perfect prediction the value of PE should be equal to 1.

The correlation function is also used to provide a comparison between the daily averaged GOES13 measurements of >2 MeV electron fluxes *F*_2MeV_ and the 1 day ahead forecasts by the REFM and SNB^3^GEO models (*F*_REFM_ and *F*_SNB_, respectively) for both the fluxes and their logarithms. For example, the correlation for the log_10_(flux) between the GOES13 measurements and forecast by the SNB^3^GEO model was defined as

$$C_{\log(\text{SNB})}=\frac{1}{N}\overset{N}{\underset{i=1}{\sum }}\frac{\left(\underset{10}{\log}(F_{2\text{MeV}}(i))-〈\underset{10}{\log}(F_{2\text{MeV}}(i))〉)(\underset{10}{\log}(F_{\text{SNB}}(i))-〈\underset{10}{\log}(F_{\text{SNB}}(i))〉\right)}{\sqrt{\mathrm{Var}(\underset{10}{\log}(F_{2\text{MeV}}))\mathrm{Var}(\underset{10}{\log}(F_{\text{SNB}}))}}$$

where *N* = 661 is the number of data points.

The prediction efficiency and correlation are common measures of the performance of a model over a long period. These measures obscure the performance of the model for the relatively infrequent but important space weather cases in which the flux exceeds a large threshold. Bodeau[2010] provides a comprehensive summary of the literature reporting anomalies caused by deep charging. Anomalies have been associated with several quantities related to deep charging such as external flux, external fluence, external current density, and internal fluence. In one study, multiple anomalies observed on a geosynchronous communication satellite were associated with 2 day >2 MeV electron external fluences between 3 × 10^8^ and 1 × 10^10^ electrons/(cm^2^ sr) [Wrenn, 1995]. This range corresponds approximately to the three daily fluence thresholds selected for the present study. For such infrequent events, the Heidke skill score (HSS) is an appropriate measure [Heidke, 1926; *Doswell et al.*, 1990; Balch, 2008]. The HSS is the ratio of the total number of correct predictions divided by the total number of observations, from both of which has been subtracted the expected number of correct forecasts by chance. Given that *w* is the number of successful negative predictions, *x* is the number of successful positive predictions, *y* is the number of false negatives, and *z* is the number of false positives, the HSS is given by [*Doswell et al.*, 1990]

(2) $$S=\frac{2(\mathrm{xw}-\mathrm{yz})}{y^{2}+z^{2}+2\mathrm{xw}+(y+z)(x+w)}$$

In the present study, a successful positive prediction is one in which the predicted daily fluence is above some threshold.

## 4 Results

Table 1 displays the resulting values of prediction efficiency PE and correlation calculated for the fluxes and their logarithms using the whole data set of forecasts/measurements. With the exception of the prediction efficiency for fluxes from REFM, all other parameters point to a very similar accuracy for the forecasts by the two models, with a marginally (≈5–10% ) better accuracy in favor of SNB^3^GEO. The prediction efficiency for fluxes from the REFM model has a negative value −1.31, indicating it to be substantially worse than the forecasts by SNB^3^GEO which has a PE = 0.6313.

**Table 1:** A Comparison of the Prediction Efficiencies and Correlations Obtained by Comparing the Forecasts of the >2 MeV Electron Flux and $\underset{10}{\log}$ (Flux) From the REFM and SNB^3^GEO Models With Measurements From the GOES 13 Satellite

Model	PE Flux	Correlation Flux	PE $\underset{10}{\log}$ Flux	Correlation $\underset{10}{\log}$ Flux
REFM	−1.31	0.73	0.70	0.85
SNB^3^GEO	0.63	0.82	0.77	0.89

**Figure 1:**
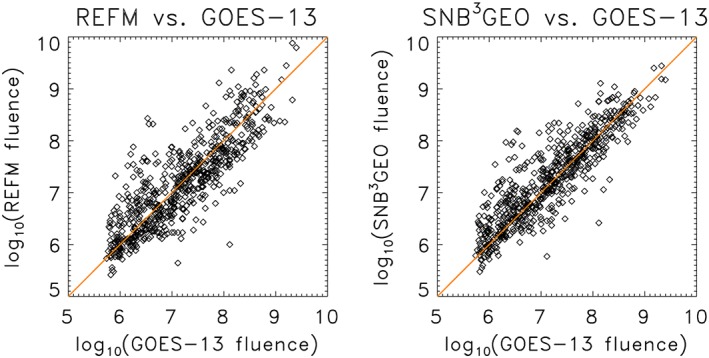
Scatterplots of (left) REFM and (right) SNB^3^GEO 1 day predictions versus GOES 13 observations for the period of interest (2 March 2012 to 31 December 2013). The diagonal is the line of perfect correlation. The lower cutoff in the observations corresponds to an instrument flux background of  10 electrons/(cm^2^ sr s).

The large difference between the PEs for *F*_2MeV_ and log_10_(*F*_2MeV_) requires some consideration of which is a better measure of model performance. The scatter plots for the two models are similar (Figure 1), with the somewhat greater scatter in the REFM results leading to slightly larger correlation values for SNB^3^GEO. The large differences in PE for *F*_2MeV_, especially the large negative PE for REFM, are dominated by the residuals due to the greatest fluences (>10^9^ cm^−2^ sr^−1^ d^−1^), which are greater for REFM than for SNB^3^GEO. In contrast, the contributions to the numerator of the PE for log_10_(*F*_2MeV_) are similar across the entire dynamic range. Since the radiation belt electron flux is approximately lognormally distributed and therefore the logarithm of flux is approximately normally distributed, the variance of log_10_(*F*_2MeV_) is a more meaningful statistical measure than the variance of *F*_2MeV_. Therefore, the PE for log_10_(*F*_2MeV_) for both REFM and SNB^3^GEO (and for any radiation belt prediction model) is the more meaningful of the two. This comparison period is short with respect to the solar cycle due to the fact that the SNB^3^GEO model archive started to operate just recently (since 2012). As such, it would be of interest to show how the PE and the correlation for the SNB^3^GEO would relate over a longer period. The only possibility is to calculate a “past‐cast” for the longer period. The model output has been calculated for such a longer period (01.01.1998–01.03.2012) with exclusion of training period (11.07.2004–11.10.2005). The measurements of the output were obtained from the Space Weather Prediction Center. Depending upon the year, measurements by GOES 9, 8, 11, 12, and 13 have been used. The resulting PE and the correlation obtained for this “past‐cast” period are PE = 0.6739 and *C* = 0.8245, correspondingly, for fluxes, while PE = 0.8187 and *C* = 0.9081 for logarithms of the fluxes. These values are very close to those in the Table 1. The value of the prediction efficiency (PE = 0.8187) is similar but seems slightly higher than the mean of the values from Figure 2 of *Perry et al.* [2010] obtained for three forecast models that were investigated by *Perry et al.* [2010].

Perhaps the most important requirement for the forecast of fluxes of relativistic electrons is to obtain accurate estimates for periods when the fluxes are high. The Heidke skill scores for the prediction of daily fluences greater than 10^8^, 10^8.5^, and 10^9^ by REFM and SNB^3^GEO are summarized in Tables 2 and 3. These tables show that during the comparison period there were 129 days with fluences >10^8^, 44 days with fluences >10^8.5^, and only 7 days with fluences >10^9^ cm^−2^ sr^−1^ d^−1^. At all three thresholds, SNB^3^GEO had better HSS. At the greatest threshold, REFM had seven false positives and three false negatives, while SNB^3^GEO had only two false positives and three false negatives. For a threshold of 10^8.5^, SNB^3^GEO had fewer false positives and negatives than REFM, while at the lowest threshold, it had many fewer false negatives though more false positives.

**Table 2:** Contingency Tables and Heidke Skill Scores for the REFM Predictions

Fluence (cm^−2^ sr^−1^ d^−1^)	>10^8^		>10^8.5^		>10^9^	
REFM HSS	0.666		0.482		0.437	
Observation	Yes	No	Yes	No	Yes	No
Forecast					
Yes	*x* = 86	*z* = 22	*x* = 23	*z* = 22	*x* = 4	*z* = 7
No	*y* = 43	*w* = 510	*y* = 21	*w* = 595	*y* = 3	*w* = 647

**Table 3:** Contingency Tables and Heidke Skill Scores for the SNB^3^GEO Predictions

Fluence (cm^−2^ sr^−1^ d^−1^)	>10^8^		>10^8.5^		>10^9^	
SNB^3^GEO HSS	0.738		0.634		0.612	
Observation	Yes	No	Yes	No	Yes	No
Forecast					
Yes	*x* = 106	*z* = 33	*x* = 31	*z* = 19	*x* = 4	*z* = 2
No	*y* = 23	*w* = 499	*y* = 13	*w* = 598	*y* = 3	*w* = 652

We estimate the sensitivity of the skill score to a single event by decreasing or increasing *x*, *y*, or *z* by 1 and accordingly decreasing or increasing one of the other variables by 1. This results in 12 unique perturbed combinations giving *S*, from which we calculate the RMS difference with respect to the actual *S*. For the threshold of 10^8^, this RMS difference was 0.66% for REFM and 0.53% for SNB^3^GEO. For the threshold of 10^8.5^, this RMS difference was 2.4% for REFM and 1.6% for SNB^3^GEO. For the threshold of 10^9^, this RMS difference was 13% for REFM and 12% for SNB^3^GEO. Clearly, the Heidke skill score is sensitive to a single event when there are very few events above a given threshold.

## 5 Discussion

**Figure 2:**
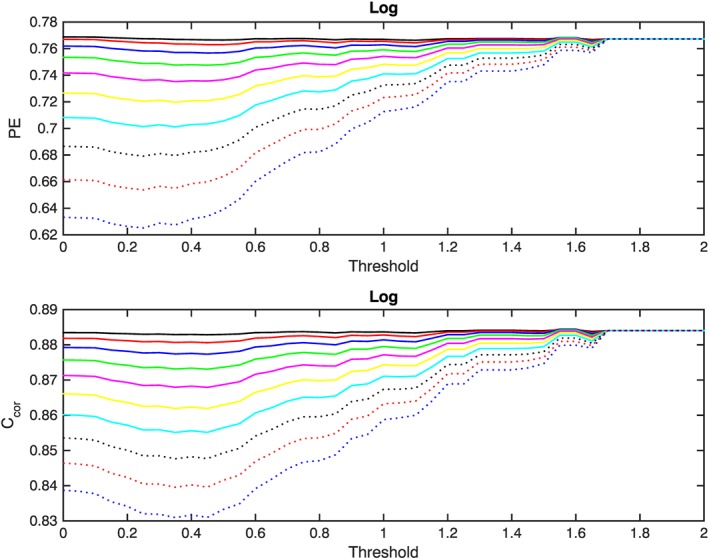
Variation of the (top) prediction efficiency and (bottom) correlation as functions of the threshold for periods when the SNB^3^GEO forecasts overshoot the measured GOES 13 values. The various lines correspond to the use of different correction factors (10% = black, 20% = red, 30% = blue, 40% = green, 50% = magenta, 60% = yellow, 70% = cyan, 80% = dotted black, 90% = dotted red, and 100% = dotted blue).

**Figure 3:**
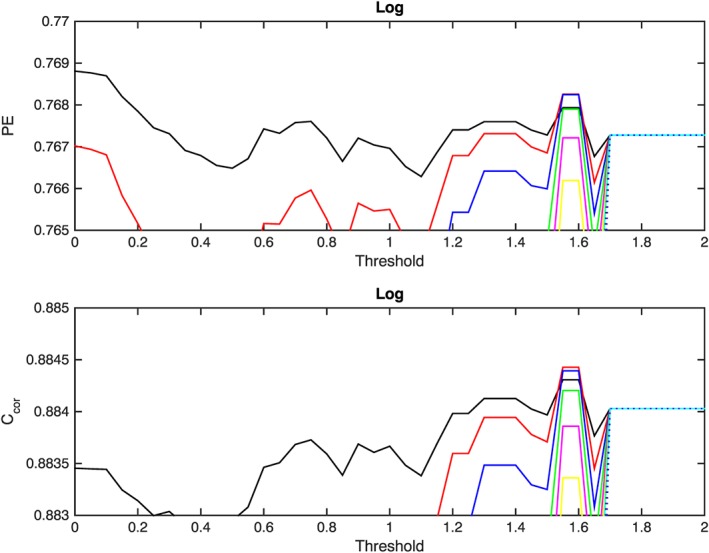
Same as Figure 2 but with a higher resolution for the *Y* axis to emphasize small changes observed in the prediction efficiency and correlation.

The parameters of the forecast accuracy presented in Tables 1, 2, 3 show that the accuracy of the forecast is very similar for the NOAA and SNB^3^GEO models with SNB^3^GEO performing slightly (5%–10%) better. One of the currently unresolved problems in the forecast of high‐energy electron fluxes at GEO with an advance time in excess of the time required for the solar wind to propagate from L1 to the magnetopause is the inability to predict dropouts caused by magnetopause shadowing [*Shprits et al.*, 2006; *Loto'aniu et al.*, 2010; *Turner et al.*, 2012, 2014]. This problem can only be solved by the accurate forecast of solar wind parameters at L1. Without such a forecast at L1 the reliability of any model to forecast the dropouts of fluxes caused by the earthward motion of the magnetopause will be very low. Moreover, in the case of the SNB^3^GEO model a significant dropout due to a large displacement of the magnetopause can affect more than one forecast value. The REFM model methodology includes a correction factor for the subsequent forecast based on recent comparison between forecasts and daily observations as it was mentioned above. In general, the incorporation of such a correction procedure into a NARMAX‐type model should not lead to a significant improvement in the performance of the model since, according to the NARMAX methodology, the error terms (*e*) of the model should account for the effects of inputs to the dynamical system that are not included within the model. In the development of SNB^3^GEO, it was found that the incorporation of error terms had little effect on the forecast quality. However, it is worth checking if some improvement in the accuracy of the SNB^3^GEO model with respect to magnetopause shadowing can be achieved by incorporating a methodology similar to that used by REFM, but in a simplified version since the time scale of magnetopause displacements is less than 1 day. This correction approach has been adopted for the forecasts of the SNB^3^GEO model in the following way. If at day *i* the value of $\underset{10}{\log}(F_{\text{SNB}}(i))$ is greater than the value measured by GOES 13 and the difference between them *D* exceeds some threshold *T* such that $D=\underset{10}{\log}(F_{2\text{MeV}}(i))-\underset{10}{\log}(F_{\text{SNB}}(i))\geq T$, the forecast for the day *i* + 1 was calculated as

(3) $$\underset{10}{\log}(F_{\text{SNB}}(i+1))-D\cdot C/100\text{\%},$$

where $\underset{10}{\log}(F_{\text{SNB}}(i+1))$ is the actual forecast by the SNB^3^GEO model for day *i* + 1 and 0≤*C*≤100% is a correction factor. Figures 2 and 3 display the resulting PE and values of the correlation for the range of threshold values 0.1≤*T*≤2. The *X* axis corresponds to the values of the threshold, while the *Y* axis represents the PE (Figure 2, top) and the correlation value (Figure 2, bottom). In both cases, the various lines correspond to the use of different correction factors (10% = black, 20% = red, 30% = blue, 40% = green, 50% = magenta, 60% = yellow, 70% = cyan, 80% = dotted black, 90% = dotted red, and 100% = dotted blue). Since the calculations have been performed using only the SNB^3^GEO model, the 10 data points that correspond to the days when the REFM forecast was not available were not excluded in these calculations.

It can be seen from Figures 2 and 3 that for threshold values greater than 1.7 (i.e., for thresholds: 1.75, 1.80,1.85,1.90, 1.95, and 2.00 ), the values of PE and the correlations do not depend upon the correction factor and threshold. This occurs because *D* is always below 1.7, implying that if the threshold level is set to a value that exceeds 1.7 the correction procedure will not be triggered, and so the values for the PE and correlation correspond to the performance of the SNB^3^GEO model without any correction. It is obvious from this figure that there is no significant increase in the values of the PE or correlation for any combination of the threshold and the correction factor. It is evident from these figures that the black and red lines, which correspond to the 10% and 20% correction factors, do not exhibit significant variations. All lines corresponding to higher correction factors indicate a significant reduction in the values of PE and correlation for lower thresholds. However, as the threshold value increases, the significance of the reduction diminishes and for values of the threshold above 1.7 all lines converge to the values of PE and correlation function without any correction. Overall Figure 2 illustrates the reliable performance of the model and shows that any additional correction will not lead to any significant improvement in the statistical characteristics of the forecast reliability. Figure 3 is the same as Figure 2 but with significantly increased *Y* axis scale to emphasize the evolution of the dynamics of the PE and correlation values that correspond to the correction factors 10% (black), 20% (red), and 30% (blue). It can be seen that for thresholds *T* < 0.2 and a correction factor of 10% the resulting prediction efficiency is slightly higher in comparison to the value without any correction but the difference is statistically negligible (≈0.15%). For the same correction factor (10%) and range of thresholds 0.2 < *T* < 1.5 the correlation function is insignificantly lower in comparison to the output of the SNB^3^GEO model without any correction. For threshold values *T* > 1.55 and correction factors below 40%, the values of the PE and the correlation function exceed the values of the uncorrected output of the SNB^3^GEO model. Statistically, the change introduced by the correction procedure is negligible and, in the first instance, it appears that the correction procedure is not able to improve the performance of SNB^3^GEO. However, let us consider the very minor improvement that the correction procedure exhibits for values of the threshold around 1.55. If the difference between the logarithms of the forecast and real measurements $\underset{10}{\log}(F_{2\text{MeV}}(i)-\underset{10}{\log}(F_{\text{SNB}}(i))$ exceeds the threshold *T* = 1.55, the predicted flux should be at least 10^1.55^≈34 times higher than that measured. For the whole period used in the comparison there were only 3 days with such a high discrepancy between the predicted and measured daily fluxes. While for the whole comparison data set the improvement in statistical parameters is negligible, it should be emphasized that this is a result of the correction applied to only 3 days from the 671 day period. Therefore, the correction that corresponds to days that follow the significant overshoot by the model may be very valuable. It is important to improve the forecast accuracy, even in cases of a very few points but with significant mismatch with the data. To quantify these improvements, the mean‐squared errors (MSE) were calculated for these three points using the SNB^3^GEO model with a zero, 10%, 20%, 30%, and 40% correction factors. Without correction MSE is equal to 0.77. The values of MSE with correction procedure implemented were 0.66, 0.61, 0.61, and 0.67 for correction factors 10%, 20%, 30%, and 40%, respectively. Therefore, for a correction factor of 20% the procedure leads to a reduction of the MSE by about 21%.

While the improvement in MSE occurs, the set of only three events cannot be used to justify the conclusion that the correction procedure has the positive effect on the forecast. Therefore, the same calculations have been done for the past‐cast period (1 January 1998 to 01 March 2012) with exclusion of training period (11 July 2004 to 11 October 2005). All together, there were 36 events for which the threshold 1.55 has been reached. Without correction MSE is equal to 0.75. The values of MSE with correction procedure implemented were 0.66, 0.65, 0.71, and 0.85 for correction factors 10%, 20%, 30%, and 40%, respectively. Therefore, for a correction factor of 20% the procedure for longer intervals lead to a reduction of the MSE by about 13%. It is worth noting that while the set of the 36 events is definitely more representative than the set of only three events, it is still too small to support the definite statement that the correction procedure improves the forecasts. However, it points out that this correction procedure has a potential to make the forecast more reliable on the days after significant forecast overshoots. The effect of applying a similar procedure on the “undershoots” of the forecast also has been investigated and it was shown that while it may improve MSE for a few events during the forecast period (2 March 2012 to 31 December 2013), the effect of the correction during the longer past‐cast period was adverse.

## 6 Conclusions

A comparison of the accuracy of the 1 day ahead forecasts of daily averaged fluxes of energetic electrons with energies in excess of 2 MeV from the Sheffield SNB^3^GEO and NOAA REFM models has been carried out for the time interval from 2 March 2012 to 1 January 2014. GOES 13 data were used to identify the accuracy of the forecast for both models. The concepts of prediction efficiency, correlation function, and Heidke skill scores have been exploited for the quantitative assessment of the reliability of the two forecasts. The main conclusion of the study is that both the prediction efficiency and the values of the correlation function, as well as the Heidke skill scores for extreme fluxes, point to the higher accuracy of the SNB^3^GEO forecast. In addition, the effect of the methodology used for forecast correction in the REFM model on the SNB^3^GEO prediction accuracy has been investigated. It is shown that while the incorporation of this methodology into SNB^3^GEO model results in a negligible increase in the overall prediction efficiency and the correlation, it may reduce errors related to the small number of data points that follow the significant overshoot for the SNB^3^GEO forecast relative to the GOES 13 measurements. The very low number of such data points explains why the subsequent reduction of errors leads to a negligible increase of the statistical forecast parameters (prediction efficiency, correlation, and Heidke skill score). However, the incorporation of the REFM methodology into the SNB^3^GEO model has the potential to reduce the effects of SNB^3^GEO significant overshoots and therefore also may improve the forecast quality.

## Supporting information



Data Set S1
